# Crawford Williamson Long and Ether*Paper read before Interstate Association of Anesthetists, at Louisville, Ky., July 26, 1916.—Reprinted from International Journal of Surgery.

**Published:** 1917-11

**Authors:** E. M. Magruder

**Affiliations:** Charlottesville, Va.


### CRAWFORD WILLIAMSON LONG AND ETHER.*

**By E. M. Magruder, M.D., Charlottesville, Va.**

### Status of Anesthesia Prior to Long's Professional Career.

There is an old adage, "Necessity is the mother of invention," and, we might add, "of discovery," as well. When

*Paper read before Interstate Association of Anesthetists, at Louisville, Ky., July 26, 1916.—Reprinted from International Journal of Surgery.

the route from Europe to India was blocked by the rise of the Mohammedan power, necessity compelled the finding of some other route. Columbus reasoned that the earth was round and that by sailing westward from Europe India could be reached, and he was right; but in the effort to reach India he stumbled upon the unknown American Continent.

Since the dawn of the profession of medicine the surgical treatment of diseases and injuries has had to be reckoned with, but the pain attending surgical operations made both patient and surgeon shrink from operative procedures.

From the day that "the Lord caused a deep sleep to fall upon Adam and took one of his ribs," efforts were constantly made to find some means of annulling pain during surgical operation, and many ingenious methods were tried; the inhalation of preparations of opium, mandrake, and cannabis indica, the administration of morphin and whiskey to dead drunkeness, the local application of cold in the form of ice, compression or large nerve trunks, compression of the common carotid arteries, bleeding till the patient fainted, etc., were some of the means employed. But none of these were satisfactory, and patients, when forced to resort to surgery, endured the agony of the knife securely strapped to the operating table, and the interior of hospitals was made hideous wiht the cries and groans of surgical victims. But the terrifying work had to go on. The great French general, Moreau, in 1813, had both legs mangled by a cannon ball and sat calmly smoking while he watched their amputation, and promptly died soon after. Compare these conditions wtih what is now going on all over the world, where we see patients begging for the luxury of an operation with no dread of the consequences.

So then the necessities of surgery urged the discovery of some means by which its horrors might be abolished, and some of the greatest minds of the scientific and surgical world set themselves to the task.

In the study of the efforts to attain surgical anesthesia and of the different steps by which it was finally reached, it is remarkable and interesting to observe how close to the goal experimenters frequently came without knowing it.

Dr. W. J. Morton, of New York, quotes Apuleis in the first or second century as saying. "If any one is to have a member mutilated, burned, or sawed, let him drink half an ounce of mandrake with wine and let him sleep until the member is cut away without pain or sensation." He also quotes Dauriol as "Claiming five cases of painless operations under the effects of Anodyne Vapors," and refers to Hickman, a London surgeon, who, in 1828, described a method of painless surgery by "The methodical introduction of certain gases into the lungs." But no mention is made of the nature of these vapors and gases.

In 1799 Sir Humprey Davey, of England, one of the greatest scientists and thinkers of the world, announced that the inhalation of nitrous oxid, which had been declared a "virulen poison," and which he found not dangerous, would produce insensibility and had cured toothache, adding these portentous words: "As Nitrous oxid appears capable of destroying pain it may probably be used with advantage during surgical operations" (Buxton). Here he stood upon the very threshold of discovery and it needed only a straw's weight to throw open the door; but Davey was not a physician or surgeon and none were found progressive enough to carry out his suggestion. The man destined to do this for suffering humanity was not born till sixteen years later, the year of Waterloo, 1815.

Again in the year 1800, William Allen, a chemist, demonstrated in the presence of the famous surgeon, Sir Astley Cooper, and others, in Guy's Hospital, London, the phenomena of nitrous oxid inhalation, noting especially the loss of sensation to pain; but the great surgeon failed to appreciate the momentous possibilities involved and the demonstration involved and the demonstration fell upon barren soil. With these two suggestions it seems that even a tyro would have grasped the plum so tantalizingly dangled before his eyes.

"Pareira of England, in his Materia Medica of 1839, stated that nitrous oxid produces pleasing delirium, desire to dance, fight, etc., and sometimes stupor, and recommended it for spasmodic asthma'' (Da Costa).

Sulphuric ether had been known for a long time, but it was regarded as too dangerous to be used. Between the year 1795 and 1839, however, different experimenters, as Pearson, Faraday, and Pariera of England, Thompson of Scotland, Godwin, Mitchell, Jackson, Wood, and Bache, of America, and Nysten, showed that the vapor of ether when inhaled would produce effects similar to those of nitrous oxid gas, such as exhilaration, excitement, laughter, crying, pugnacity, and insensibility, would relieve the spasmodic disorders of colic, asthma, whooping cough, catarrh, phthisis, and chlorine gas, and would produce anesthesia or insensibility to pain.

For a number of years, then, in spite of the suggestions of Davey and Allen, the surgical profession still paused at the threshold of discovery. As late as 1839 Velpeau of Paris wrote: ''To escape pain in surgical operations is a chimera which we are not permitted to look for in our day'': and, in 1846, Sir Benjamin Brodie said: ''Physicians and surgeons have been looking in vain from the day of Hippocrates (460 B. C.) down to the present time for the means of allaying or preventing pain'' (Buxton). Brodie had not then heard of Long's discovery.

Leaving behind now the dignified, painstaking research of thinkers, scientists, and experimenters, we come to the comic part of the drama and find that a queer, amusing, and even frivolous custom, was due to the grandest discovery of the universe.

During the first half of the 19th century there was an odd class of persons who wandered about the country, first in England and then in the United States, lecturing in public about chemistry. During the course of the lecture it was the custom to invite certain members of the audience upon the stage to inhale nitrous oxid or ether vapor ''for the purpose of provoking exhilaration, excitement, semi-conscious gyration, and mirth-provoking antics, for the amusement of the spectators.'' This first began with the use of nitrous oxid, but soon ether became the favorite for the reason that it was more easily manipulated (Buxton).

These strange entertainments were called ''Nitrous Oxid or Ether Frolics,'' and soon became fashionable at private social gatherings.

It was in connection with these apparently silly performances that the discovery of surgical anesthesia was made, by the side of which Columbus's discovery of America, Newton's discovery of the law of gravitation, Watt's discovery of the expansive force of steam, and Fulton's invention of the steamboat, are compelled to take second place. These added to the material wealth of the world, which surgical anethesia relieves human suffering and saves human life.

The first man to use a known anesthetic in an authentic operation was Dr. Crawford Williamson Long of Jefferson, Georgia, the date being March 30, 1842.

Let us now consider the life history of the man who has brought the greatest blessing to mankind.

**Sketch of Crawford Williamson Long.**

Crawford Williamson Long, son of James Long and Elizabeth Ware, and named for Georgia's great statesman, William H. Crawford, the friend of his father, was born at the little town of Danielsville, Madison County, Georgia, November 1, 1815, the year of Waterloo and Napoleon's overthrow.

His paternal grandfather, Samuel Long, came from Donegal County in the north of Ireland and settled at Carlisle, Pennsylvania, in 1762. After serving with Washington and LaFayette through the Revolutionary War, he moved with his family to Madison County, Georgia, in 1792, taking with him his eleven-year-old son, James, who had been born at Carlisle. This james Long, father of Crawford, married Elizabeth Ware, a Virginia girl, whose parents were born and reared in Old Albermarle County, Virginia (my own native county), and had emigrated to Georgia and settled in Madison County.

It will thus be seen that Crawford W. Long came of that sturdy North of Ireldan, Scotch-Irish stock, from which sprang some of America's greatest soldiers and statesmen, as Andrew Jackson, Henry Clay, William H. Crawford, Stonewall Jackson, Abrahma Lincoln, and others—a race that held Ireland for the house of Orange against odds of six to one, that overcame the savage at Point Pleasant and the British at King's Mountain and New Orleans, that was chiefly instrumental in winning American independence, and that for a century was a living wall of protection between the settlements on the Atlantic slope and the western Indians.

It is said that the North Carolinians with their slogan: "First at Bethel, farthest to the front at Gettysburg, and last at Appomatox," are the best claimers in the world; but the Virginians are a close second, for we feel perfectly sure that it was Dr. Long's maternal Virginia blood that enabled him to make his grand discovery.

When 14 years old young Long attended Franklin College, now the Universtiy of Georgia, at Athens, where he took the degree of Master of Arts at the age of 19, in 1853, being considered "studious and wise" beyond his years and called "The Baby" at college on account of his extreme youth. He stood second in the graduating class. His room mate and best friend at Franklin College was Alexander H. Stephens, afterwards Vice-President of the Confederacy. Long then taught for one year in the Danielsville Academy, and after that began the study of medicine at Jefferson under Dr. Grant and took a medical course of one year at Trasylvania University, Kentucky (this fact gives Kentuckians also a claim upon the discoverer of surgical anesthesia). In the fall of 1837 he entered the University of Pennsylanvia at Philadelphia as a medical student and graduated in medicine in 1839, in two years, at the age of 23 years. He then went to New York and spent 18 months "walking the hospitals," specializing in surgery, and witnessing much suffering therefrom; he there attained a reputation as a skillful surgeon. While in the North, Long, with other students, indulged in "Ether Frolics" in his room at his boarding house and thus became familiar with the effects of ether.

In 1841, when 26 years old, he settled in the little town of Jefferson, Jackson County, Georgia, buying out the practice of his old preceptor, Dr. Grant "soon acquiring an extensive and lucrative practice," which grew with the years and extended into the neighboring counties and town of Georgia. His house in the village became a favorite social resort for the young men of the neighborhood and the students of Jefferson Academy, of which W. H. Thurmond was the principal, and it was here that the great discovery was made, March 30, 1842.

On August 11, 1842, Dr. Long married Mary Caroline Swain, the sixteen-year-old daughter of a planter, George Swain, and niece of Governor David L. Swain of North Carolina, who was afterwards president of the University of North Carolina, a lovely and intellectual woman, fit in every way to be the life partner of such a man. She was born December 14, 1825, and died September 22, 1888, at Comfort, Texas, from injuries received in a railway wreck. She lies buried with her noble husband and children in Oconee Cemetery at Athens, Georgia.

The children born to this marriage were nine, as follows:

Henry, who died at the age of one year; Sarah Frances, who married Dr. M. E. Taylor (U. S. A.); Edward Crawford, who married Cora C. Stroud; Lizzie, who died when fourteen months old; Ella, who lived to be 8 years and 9 months old, and died of scarlet fever; Florence Cornelia, who married Col. J. L. Bartow (C. S. A.); Eugenia Anna, who married Hon. A. O. Harper, Arthur B., and Emma M.

During the year 1850 Dr. Long resided in Atlanta, which was then in such a crude stage that, desiring better educational and social advantages for his children and also with the desire to be near his parents and his brother, who intended making Athens their home, he moved, in 1851, with his family to that city which had a population of two or three thousand and was larger than Jefferson and about 20 miles from the latter. In Athens he spent the remainder of his useful life and there built up a "heavy practice," especially in obstetrics and surgery and in consultation work, becoming most popular with the medical profession on account of his high profesional attainments and absolute loyalty to his brother practitioners. He was often summoned long distances to assist in difficult operations and patients came to consult him from other State, as Alabama, Florida, etc.

It is said that he cured several cases of lockjaw, advocated and used fresh air and abundant diet in tuberculosis, ether in obstetrics, and was especially skilled in the use of obstetrical forceps. In a very large obstetrical practice of 36 years' duration he is credited with only two deaths, one being due to fright after an electrical storm.

Long was reared a Presbyterian, his father and paternal grandfather having been elders in the Presbyterian Church, but he joined the Methodist Church in order to be with his wife. He considered slavery as God's method of civilizing the African and felt deeply the responsibility of having slaves to control and influence. He owned two plantations with an "overseer" for each, who were told that under no circumstances was a slave to be severely punished. Nearly all his former slaves remained on the plantations after they were freed until some years after his death. Politically he was a Whig, and, with his friend, Alexander H. Stephens, opposed secession; but like many other patriots he "went with his state."

During the Civil War he was never in active service, having been appointed by the ConfederateGovernment surgeon in charge of the Confederate Hospital on the University Campus at Athens and of the families of Confederate soldiers fighting for "The Cause" The Southern Cross of Honor was arranged for him in 1912 by the United Daughters of the Confederacy.

Dr. Long received pardon (?) for participationg in the so-called "Rebellion" from President Andrew Johnson, the warrant of pardon being dated October 2, 1865, and received September 10, 1866, and on May 28, 1867, he was made Acting

Assistant Surgeon of the United States Army for the Union troops stationed at Athens, with a salary of forty dollars a month. Such was his reputation as a man and a surgeon that no oath of allegiance to the United States Government was required and he was thanked for his humanity and skill.

Before the Civil War Dr. Long was a wealthy man, but lost everything by that struggle, and, in 1866, with reference to pushing his claims to the discovery fo surgical anesthesia before Congress, he wrote to Hon. David L. Swain: "It is all I can do to obtain funds for the necessaries of life. Under these circumstances I will remain quiet for the present." While he never attained, after the war, his former financial status, he derived a comfortable support from his practice and died in comfort, June 16, 1978, at the age of 62, in Athens, Georgia, where he had lived and worked since 1851, and where he now lies buried.

He was stricken with apoplexy, due to overwork, and became unconscious at the bedside of a patient, the wife of his friend, Dr. H. H. Carlton, whom he was attending in Confinement. He suddenly fell over on the bed unconscious and was carried to another room in Dr. Carlton's house where he died the next day. On regaining consciousness, which he did before his death, he asked, "How is she?" and said, "Care for the mother and child first," at tthe same time giving the directions for treatment.

Long's portrait hangs in numerous colleges and hospitals and in the Anesthetits' Hall of the Royal Society of Medicine in London; a life sized portrait of him, the gift of Henry Stuart of New York, may be seen in the Capitol of Georgia.

The Medical Association of Georgia, in 1910, unveiled to his memory a granite monument in the form of a shaft at Jefferson, near the spot on which the first operation with ether was performed; this monument was donated by Dr. L. G. Hardman, of Georgia, to the Medical Association of Jackson County, Georgia.

The infirmary connected with the University of Georgia is a Long memorial.

In 1912 the University of Pennsylvania, his Alma Mater, unveiled in its medical building a bronze medallion with the inscription:

To the Memory
Of
Crawford W. Long
Who First Used Ether as an
Anesthetic in Surgery
March 30, 1842

The State of Georgia, through her Legislative Committee on Appropriations, has already signified her intention to place a statue of Crawford W. Long in Statuary Hall, Washington, D. C., along with that of his friend, Alexander H. Stephense, as soon as the finances of the state warrant an appropriation for the purpose.

It was Stephens who, in 1878, soon after Long's death, when asked to suggest the names of two of Georgia's famous men for Statuary Hall, suggested those of Long and Oglethorpe.

**Personality and Character of Long.**

Dr. C. W. Long stood six feet high and had a fine physique, broad shoulders, small feet and hands, and a most delicate touch. He sometimes engaged in dancing, loved games, and played whist with his family, being a fine player, but he never approved of poker or seven-up. He was also fond of hunting and fishing and was a good swimmer and noted for his activity, being able to spring upon his horse without touching the stirrup. Exceedingly intellectual and fond of books he was an omnivorous reader and delighted especially in Shakespeare, Scott, Byron, Burns, and other classics.

Dr. Long was proud and sensitive; he never offered an insult, but it was well known that he would never take one. His friend, Judge Cobb, used to say there was nothing of which Long was afraid, but he would always dodge a bee. On one occasion, soon after the Civil War, when Athens was garrisoned by federal soldiers, as Long was returning home he heard screams in a nearby house, from which soon isued a woman and child pursued by a drunken federal soldier; instantly dismounting Long jerked a paling from the yard fence and administered a severe beating to the drunken man, although the latter was heavily armed. He was sometimes satirical. In a conversation with friends one day he used the expression, "An aching void." A "Smart Alec," who was present, said, "Doctor, how can that be? No void can ache"; to which Long calmly replied, "Have you ever had the headach?" The quaint expressions of the ignorant were an unfailing source of amusement to him, though he was too careful of their feelings to allow them to see it. He once received a note from a patient: "Doctor, please come and see me; I am very sick; when I stands up I falls down, and when I goes to sleep I lies awake all night." When urged to take a rest he would say: "My sick need me." He was loved by both white and black, the latter often speaking of him as "Doctor Savior."

Those who knew Dr. Long most intimately testify to the fact that he was "a man, and that nothing that pertains to a man was foreign to him," and his work shows that he had the courage of his convictions.

One of the rules of his life was, "If I cannot say a good word for a person I will say nothing."

Some of his friends urged him, soon after his graduation in medicine, to enter the United States Navy; but he refused in order to be near his old father.

At the funeral service of Dr. Long held in Athens, the Rev. A. A. Lipscomb paid the following tribute to his memory: "One of the lessons of Dr. Long's life is that worth will make its way to the admiration and reverence of mankind. Postponed it may be, but when the honor is attained it never fails to repay the tardiness of time and compensate the lagging march of circumstances.

"Dr. Long assumed nothing, pretended to nothing, was thoroughly truthful, and walked humbly before God. He was modest to timidity but stern and bold when danger confronted.

"Reticent as to his own merits, reticent of his troubles lest he should disturb the happiness of others, he kept the covenant of a heart's true life till his days were numbered, a pattern of a Christian physician." Of his life most appropriate are the words: "That best portion of a good man's life, his little, nameless, unremembered, acts of kindness and love."

**Professional Standing of Dr. C. W. Long.**

In 1858 Dr. Long was recommended for a professorship in a medical faculty, and the following testimonial by Dr. I. J. M. Goss, of Jefferson, Georgia, will show his standing with the medical profession: "You could not select a more suitable man from New Orleans to New York than Dr. C. W. Long. He is in every way the man to fill a professorship—a man of extensive capital, of profound erudition, of mature medical knowledge, of most amiable deportment, of high toned felings, and of noble bearing; a man that everybody esteems, an eminent practitioner, a surgeon of as high respect as any of his age in the South, unusually successful, unassuming, like Washington, content with deserving a crown but refusing to wear it, of great energy and point of character, even tempered, and gentlemanly.

"His opinion is sought far and wide by the profession; people are satisfied with any physician if he but procures Dr: Long's opinion; a profound medical philosopher with no hibbies, always up with improvements of the profession, one of the best pathologists in the South, he stands unrivaled in each department of his profession."

The physicians of Athens after his death passed the following tribute to him:

"Dr. C. W. Long was an honor to the profession, regarding it as a medium through which to make his life a blessing to the world. He was a high minded Christian gentleman, always just and liberal towards his professional brethren, holding sacred their reputation as his own by strictly observing the highest code of medical ethics in all his association with them. He was never heard to make reflections or criticisms detrimental to any with whom he called in consultation. All his neighboring practitioners held him in the highest esteem and confidence, and almost invariably Dr. Long was called to attend sick physicians and their families.

"His sympathy for woman was always manifest in his self-sacrificing devotion for her relief and comfort in the hour of trial and suffering, as was so nobly displayed in the very last act of his life; nor would he ever refuse his skill even to the porest negro woman."

We come now to the great discovery that helped to make Long famous and of which he was most proud.

**The Great Discovery.**

The discovery of surgical anesthesia may justly be regarded as the greatest achievement of the human race, and the man who accomplished it deserves the highest praise. But he to whom such honor is accredited should have his claims rest upon foundation so firm as t oleave no doubt as to their justice.

It is not disputed that sulphuric ether was the first authentic agent successfully used as an anesthetic in surgery, and it has been proved beyond the least doubt that the late Dr. Crawford W. Long of Georgia is entitled to claim priority and publicity in the discovery and use of sulphuric ether as an anesthetic in surgery.

The foundations for Dr: Long's claims are:

1. His own undeniable statement. It has already been known that Dr. Long was a perfectly honest, honorable, and reliable man, and worthy of all credence.

2. Affidavits signed by the patients upon whom he operated, by the relatives and friends of his patients, and by eye witnesses of his operations. These affidavits, made by persons certified by the proper officials as honest and reliable, were subscribed before justices of the peace and notaries public, and they are now in the possession of Dr. Long's two daughters living in Athens, Georgia, who cherish his memory and guard his interests with the courage of true daughters of Sparta; and it is from these affidavits and from the private correspondence of Dr. Long and other literature kindly loaned me by his noble daughters, that the facts regarding Long and his work have been derived, and every statement made with regard thereto can be proved.

On March 30, 1842, Long made the discovery of surgical anesthesia; in 1848, he presented the account of his discovery before the Faculty o fthe Medical College of Georgia at Augusta; in December, 849, he published in the Southern Medican and Surgical Journal of Augusta his first paper upon the same subject, and in April, 853, he read a similar paper at Savannah before the Medical Association of Georgia (founded in 1849), which, after a committee investigation, unanimously passed the following resolution:

"Resolved, that it is the opinion of the Society that Dr. Crawford W. Long was the first person who used sulphuric ether as an anesthetic in operations and we recommend him to present at once his claims of priority in the use of this agent to the consideration of the American Medical Association at its next meeting."

The following is Long's own verbatum account of his great discovery taken from his first paper:

"Previous to stating the first operation performed with ether I will briefly give the reasons which induced me to make experiments in etherization.

"In the month of December, 1841, or January, 7842, the subject of the inhalation of nitrous oxid gas was introduced in a company of young men assembled at night in the village of Jefferson, Georgia, and the party requested me to prepare them some. I informed them that I had not the requisite apparatus for preparing or using the gas, but that I had an article, sulphuric ether, which would produce equally exhilarating effects and was as safe. The ether was introduced and all present inhaled. They were so much pleased with its effects that they afterwards frequently used it and induced others to do the same, and the practice soon became quite fashionable in the country and some of the contiguous counties.

"On numerous occasions I inhaled ether for its exhilarating properties, and would frequently at some short time subsequent to its inhalation discover bruises or painful spots on my person, which I had no recollection of receiving and which I felt satisfied were received while under the influence of ether. I noticed my friends, while etherized, sustain falls and blows which, I believed, were sufficient to produce pain on a person not in a state of anesthesia, and, on questioning them, they uniformly assured me that they did not feel the least pain from these accidents." (One young man received an injury of the ankle that disabled him for several days.)

"Observing these effects I was led to to believe that anesthesia was produced by the inhalation of ether and that its use would be applicable in surgical operations."

In a letter to Governor Swain, Long says: "I cannot give the exact date I was first led to believe that sulphuric ether (its inhalation) would prevent pain in surgical operations, but know it was as early as February, 1842." According, however, to Dr. Long's daughters, the above first mentioned "Ether Frolic" occurred during the last week in December, 1841, Christmas week, being in Dr. Long's office and the first of its kind ever held in Georgia. It was during this week that he confided to his friend, R. H. Goodman, his belief in the anesthetic powers of ether in surgery, and in the following February he decided to use it at the first opportunity. The following letter from Dr. Long to his friend, Robert H. Goodman, who moved from Jefferson to Athens, January 20, 1842, and intreduced "Ether Frolics" there, may be of interest:

Jefferson, Ga.,
February 7, 1842.

Dear Bob:

I am entirely out of ether and want some by tomorrow night. We have some girls in Jefferson who are anxious to see it taken, and you know nothing would afford me more pleasure than to take it in their presence and get a few sweet kisses, etc.

Your friend,

C. W. Long.

Goodman states that this was the order for the ether with which Long performed his first operation on a patient under the influence of that drug.

Long's paper continues:

Case 1. "The first person to whom I administered ether in a surgical operation was James M. Venable (21 years old), March 30, 1842, who then resided within two miles of Jefferson, Georgia (and attended the Jefferson Academy).

"Mr. Venable consulted me on several occasions in regard to the propriety of removing two small tumors situated on the back of his neck, but he would postpone from time to time having the operation performed from dread of pain.

"At length I mentioned the fact of my receiving bruises while under the influence of the vapor of ether (at 'Ether Frolics') without suffering, and, as I knew him to be fond of and accustomed to inhale ether, I suggested to him the probability that the operation might be performed without pain, and proposed operating on him while under its influence. He consented to have one tumor removed, and the operation was performed the same evening. The ether was given to Mr. Venable on a towel and, when fully under its influence, I extirpated the tumor. It was encysted and about half an inch in diameter. The patient continued to inhale ether during the time of the operation and, when informed it was over, seemed incredulous until the tumor was shown to him.

"He gave no evidence of suffering during the operation and assured me after it was over that he did not experience the least degree of pain from its performance."

(This was the first authentic surgical operation ever performed with a known anesthetic, and, as the discovery of America unexpectedly occurred in the effort to achieve another goal, so likewise the discovery of surgical anesthesia was unexpectedly accomplished in connection with an entirely different object— the pursuti of pleasure in "Ether Frolics.")

Case II. "The second operation I permored on a patient etherized was on the 6th of June, 1842, and was on the same person for the removed of the other small tumor. This operation required more time than the first, from the cyst of the tumor having formed adhesions to the adjoining parts. The patient was insensible to pain until the last attachment of the cyst was separated, when he exhibited signs of slight suffering, but asserted after the operation was over that the sensation of pain was so slight as scarcely to be perceived." (Witnesses, Wm. H. Thurmond, A. T. Thurmond, E. L. Rawls and J. E. Hayes; see their affidavits.)

Case III. "My third case was a negro boy (Jack) who had a disease of a toe which rendered amputation necessary, and the operation was performed July 3, 1842, without the boy evincing the slightest degree of pain."

Case IV. "From Mrs. Mary Vinson, September 9, 1843, I removed three tumors of the scalp the same day. The inhalation of ether was used only in the second operation and was effectual in preventing pain, while the patient suffered severely from the extirpation of the other tumors." (Witnesses, Wm. Vinson, husband of the patient; see his affidavit.)

Case V. "On January 8, 1845, I amputated two fingers of a negro boy (Isam). The boy was etherized during one operation and not during the other. He suffered from one operation (the one without ether) and was insensible during the other (the one with ether). The reason the second amputation was done without ether was to make sure that the freedom from pain in the first was due to the ether and not to imagination." (Witnesses, G. L. Thompson, Milton Bailey and J. W. Groves, the latter the first medical student of Dr. Long, who assisted in this operation; see their affidavits.)

Case VI. In 1844, Dr. Long adminisiered ether to a small boy, J. W. Glenn, and extracted one of his "baby teeth" without pain (affidavit of J. W. Glenn).

Case VII. In the summer of 1846 he painlessly extracted a tooth from Mrs. Mary E. Ware while the latter was under the influence of sulphuric ether. Witness, Mrs. Eliza Nabers; see her affidavit.)

While awainting cases for operation Long kept up his study of the effects of ether, as the following statement of dosage and other particulars of the treatment are covered in Parke, Davis & Co.'s literature on Typhoid Phylacogen.

Dr. Groves, his first medical student (1844-1845), will show:

"We often, with closed doors, would retire to the back room of the office, and he( Long) would administer it to me, and then after giving me certain cautions would allow me to administer it to him, to prove its perfect anesthetic effect and also to discover any bad effect."

It is a curious fact that several of the world's great scientific discoveries were made nearly the same time by different men in widely separated localities and without the knowledge of each other's achievement. Thus oxygen was discovered by Priestly of England and Scheele of Sweden independently of each other the same year, 1774; and, during the years 1842 to 1846, incusive, three men independently discovered surgical anesthesia.

Dr. Crawford W. Long, a physician of Jefferson, Georgia, made the discovery first, with ether, in surgery, March 30, 1842, as just related.

Dr. Horace W. Wells, a dentist, of Hartford, Connecticut, came next, with nitrous oxid in dentistry, Decemmber 11, 1844, two and a half years after Long's first operation. On the preceding day he had seen Colton, a wandering lecturer, administer nitrous oxid in Hartford to a man on the stage who sustained a severe fall and injured his legs without pain. On the next day Wells had Colton administer the gas to him while a brother dentist painlessly extracted one of his sound molar teeth, and the same year he successfully used it on 12 or 15 patients, and it was employed by other dentists in Hartford.

In January, 845, and again in 1847, Wells attempted a public demonstration of the anesthetic effect of nitrous oxid in the Massachusetts General and New York Hospitals, but his attempts were unsuccessful as the patients seemed to suffer as much pain as without it. These failures so discouraged Wells that he soon abandoned his discovery.

Dr. Wm. Thomas G. Morton, a dentist of Boston, Massachusetts, came last, with ether in dentistry, September 30, and in surgery October 16, 1846, four and a half years after

Long's experiment. While attending medical lectures at Harvard he had seen a man in an "Ether Frolic" sprain his ankle without pain. He was also acquainted with Well's experiments. After producing insensibility in himself with ether he consulted Jackson with regard to the best agent to prevent pain in dentistry, and Jackson gave him a mixture of ether and aromatic oils (the latter bing added to disquise the odor of ether in order to prevent its recognition), which Morton used successfully in his office in extracting a tooth, September 30, 1846, the patient's name being Eben Frost. After being informed by Jackson what the mixture contained he and Jackson patented it under the name of "Letheon," and, on October 16, Morton administered it in the Massachusetts General Hospital to a young man, named Gilbert Abbott, while Dr. J. C. Warren painlessly removed a vascular tumor of the neck, in the presence of many physicians and the medical students of Harvard.

On the next day Dr. Georg Hayward in the same hospital removed a fatty tumor from the arm of a woman anethetized by the same agent. It was then learned that "Letheon" was patented, and the surgeons of the hospital refused to use it further until its nature was disclosed, which was done by Morton November 7, 1846.

Two other operations were now performed under its influence, amputation of the thigh by Dr. Geo. Hayward, and removal of a portion of the upper jaw by Dr. J. C. Warren, and on November 18, 1846, Dr. Henry L. Bigelow of Boston published a paper upon these operations in the Boston Medical and Surgical Journal, which was the first printed publication on surgical anesthesia ever made. The news of the discovery then rapidly spread over the world and it was at once universally adopted, being used in London and Paris in December, 1846.

All three of these experimenters did practical work and claimed the credit of the discovery.

In addition to these Dr. Chas. T. Jackson, an analytical chemist of Boston, Massachusetts, put in claims on the ground that, late in February, 1842, he had been relieved of difficult and painful breathing by inhaling ether (an alread well-known remedy for that trouble), and had thought that it might answer as an anesthetic in surgery (though he did not suggest it to any medical man till 1843, when no notice was taken of it), and that in September, 1846, he had suggested its use in dentistry to Morton, though he never, himself, did ny experimental work in that line.

(To Be Continued)

